# Improving timeliness and completion of infant vaccination among infants in Nigerian urban slums through older women's participation

**DOI:** 10.3389/fpubh.2022.898636

**Published:** 2022-09-08

**Authors:** Folusho Mubowale Balogun, Eniola Adetola Bamgboye, Abimbola Ellen Akindolire

**Affiliations:** ^1^Institute of Child Health, College of Medicine, University of Ibadan, Ibadan, Nigeria; ^2^Institute of Child Health, University College Hospital, Ibadan, Nigeria; ^3^Department of Epidemiology and Medical Statistics, Faculty of Public Health, College of Medicine, University of Ibadan, Ibadan, Nigeria; ^4^Department of Pediatrics, Faculty of Clinical Sciences, College of Medicine, University of Ibadan, Ibadan, Nigeria

**Keywords:** infant vaccination, vaccination timeliness, urban slums, older women, randomized experimental study

## Abstract

Nigerian urban slums have a high population of infants with suboptimal vaccination despite previous interventions. Older women traditionally play supervisory roles in infant care in Nigeria but their influence is untapped in infant vaccination. This study sought to determine if training of older women (≥35 years) in urban slum communities in Ibadan, South west Nigeria, and involving them in infant vaccination will improve infant vaccination timeliness and completion. This was a randomized experimental community study and pregnant women in their third trimester, residing in seven urban slum communities were randomized using their antenatal clinics (ANCs) into intervention (six ANCs) and control groups (six ANCs). The older women who will supervise the care of the infants of pregnant women in the intervention group had seven sessions of training on the importance of infant vaccination timeliness and completion. The vaccinations of the infants from both groups were compared from birth till 9 months. Data were analyzed using descriptive statistics and Chi square test at α = 0.05. There were 96 older women, 198 pregnant women (105 in intervention group and 93 controls) and 202 infants (109 in intervention group and 93 controls). Infants in the intervention group (67.9%) significantly had both timely and complete vaccinations compared with those in the control group (36.6%). Vaccines given at birth were the least timely in both groups. More infants whose older women caregiver were married had timely and complete vaccinations. Also, a higher proportion of male infants, low birth weight babies and infants with older women caregiver with at most two children had timely and completed vaccinations but these were not statistically significant. Training of older women caregivers improved infant vaccination timeliness and completion in these urban slum communities. This model may improve infant vaccination in other similar urban slum settings.

## Introduction

Despite the efforts to improve the uptake of infant vaccination in the last decade in Nigeria, the country was still one of the 10 countries whose infants constituted 60 percent of unimmunized or partially immunized infants globally in 2020 (1). According to the Nigerian National Demographic Health Survey report of 2018, basic infant vaccination uptake among children aged 12 to 23 months improved from 23 percent in 2008 to 31 percent in 2018 while those in the same age group who were never immunized reduced from 29 percent to 19 percent over the same period (2). This is not an impressive improvement and calls for a review of the current interventions which are being used to improve infant vaccination uptake. Apart from the generalized efforts aimed at the promotion of infant vaccination uptake in Nigeria (3), specific strategies are required to target particular sections of the population where infant vaccination uptake remain low.

Infant vaccination uptake has been shown to be least among infants from families from the least wealth quintiles in Nigeria (2, 4). While this category of infants is commonly assumed to be more in the rural areas, a multicountry study showed that living in urban area was associated with incomplete infant vaccination in sub-Saharan Africa (5). A significant number of these infants live in urban slums (6) and they are usually infants of mothers with little or no education (a common characteristics of most unimmunized infants in Nigeria) (4). The segregation of infant vaccination data into urban and rural prevents the appreciation of differences in the vaccination coverage between the different socioeconomic strata in urban areas. However, the uptake of health interventions, including vaccination have been shown to be worse among urban slum children (7, 8) and this can be responsible for the poorer under five health indices seen in these areas compared with the rural areas and formal urban settings (9–11). Earlier literature has suggested that the peculiarities of urban slums need to be considered in designing strategies aimed at improving vaccination uptake among urban slum infants (12). This will not only provide a short-term solution to the problem of infant under vaccination, but it will have a long term effect on under five health indices and ensure that infants in urban slums are not left behind in the effort to improve infant vaccination uptake in Nigeria.

Older women are known to play important roles in infant care in developing countries and they have great influence in decision making about uptake of infant health care services (13–15). Some of these influences have been negative as they have been reported to frustrate efforts at compliance with exclusive breastfeeding of young infants (15) and in the prevention of mother to child transmission of HIV (16). However, there were instances where the influence of the older women were positive like improved timeliness and completion of vaccination of impoverished infants who were living with their grandmothers in Indianapolis, United States (17). The influence of the older women can be harnessed in ensuring optimal infant vaccination uptake in urban slums in developing countries and this can improve the health indices of these children in the long term.

For an optimal infant vaccination to be realized, both the timeliness of the vaccine and the completion of the schedule are equally important. Many epidemiological data emphasize infant vaccination coverage without taking into cognizance the timeliness of the vaccine. Vaccines are scheduled based on the age the infant will be able to mount adequate immune response to the antigen being introduced and become immunized (18). This schedule is also made to coincide with the time that the maternal antibodies that were transferred in pregnancy begin to reduce (19). Failure to immunize infants as scheduled makes them not only vulnerable to vaccine preventable infections, but it also threatens the development of herd immunity in the community and expose other infants to infections as well. Infants who delay vaccines are also more likely to have incomplete vaccination (20). The problem of delayed and incomplete infant vaccinations has been described in earlier studies (21, 22) and it is important to take a further step by addressing this problem, specifically in the context in which it occurs.

The culture of older women overseeing the care of infants is being practiced in urban slums in Nigeria and their influence in infant care decision making can be used in improving infant vaccination among infants in families living in urban slum. It is on this premise that this study aimed to determine the differences in the infant vaccination timeliness and completion among infants whose older women carers were trained about the importance of infant vaccination and those who were not trained.

## Methods

### Study design

This was a randomized experimental community study in which pregnant women in their third trimester were randomized into intervention and control groups.

### Study area

The study was conducted in seven urban slum communities which spread over Ibadan North and Ibadan Northeast local government areas in Ibadan, a city in southwest Nigeria. Most of the inhabitants were of Yoruba origin and culturally, older women (including relatives like grandmothers, Aunties and non-relatives like neighbors) oversee the care of infants and as a result are very influential in decision making about infants' care. Infant vaccination services in the study communities were being provided by 10 Primary Health Care (PHC) centers and a secondary health center (which had two immunization clinics) in the communities as at the time of this study. These health centers also provide antenatal care services for pregnant women. There were also antenatal clinics which were run by community birth attendants who were under the supervision of the primary health care centers. These community birth attendants were resident in the communities and trained by the State Ministry of Health to provide basic midwifery services to pregnant women in the communities. Infants delivered at these clinics were being referred to the PHCs for vaccination.

### Study population

These were infants whose mothers lived in urban slum communities and had antenatal care at the health facilities located in these communities.

### Study participants and sampling

The mothers of infants who participated in this study were recruited in their third trimester from all the 10 PHCs and two immunization clinics hosted by the secondary health center that serve the communities by trained research assistants. A sample size was not calculated a priori because the proportion of the population of women who live in urban slums that access healthcare services in the health facilities was not known due to their poor utilization of the services. The definition of urban slum was according to the UN-HABITAT criteria (23) which defined a slum dwelling as any residential house in which any of the following criteria that is required for good housing is lacking:

1. Durable house with a permanent structure that can protect its inhabitants against extreme climate.
2. Enough space with a maximum of three people living in one room.
3. Availability of affordable and adequate safe water for use.
4. Good sewage disposal which may be privately owned toilet facility or public toilet that is shared by a reasonable number of people.

**Figure 1:**
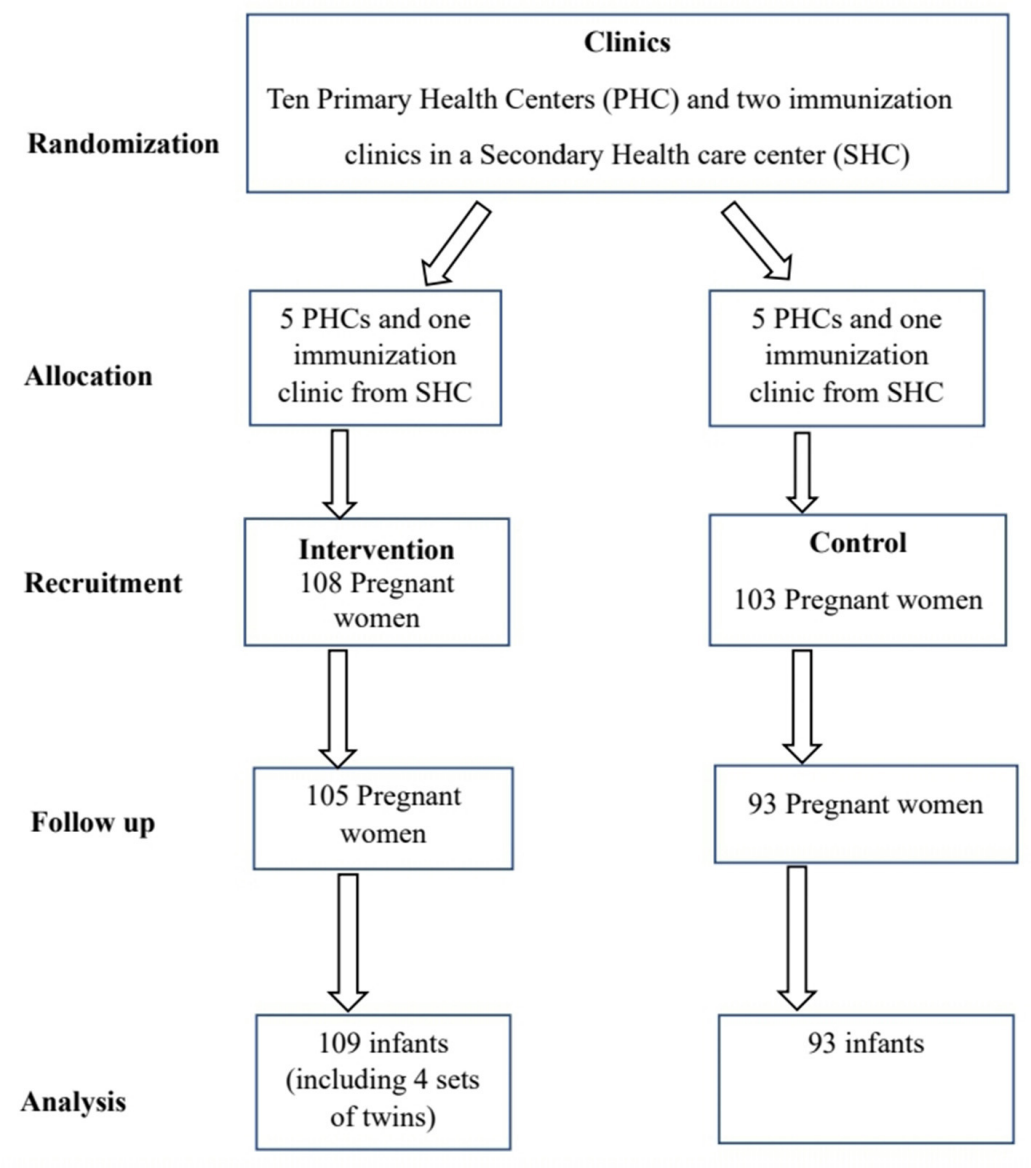
Flow of study participants.

Women who reported that their residences fulfilled these criteria were recruited and a visit was made to validate the information provided. Figure 1 shows the flow of the participants all through the study. The women were then recruited consecutively over a 3 month period (November 2018 to January 2019), and randomized using their ANCs into intervention group (five PHCs and one clinic in the secondary health center) and the control group (five PHCs and one clinic in the secondary health center) as shown in Figure 1. This was achieved through balloting by FMB. The women in the intervention group were asked to bring the potential older woman caregiver for their unborn babies to the clinic.

### The study intervention

These women were trained using a seven-module manual on the importance of infant vaccination, timeliness and completion of the schedule. The manual was developed based on findings from focus group discussions among women of similar age group from these communities. Participatory learning method was used and each training session lasted for about 90 to 120 min. Each module had assessment strategies to monitor the knowledge gained from each training session. Further details about this training manual, the training process and the effect on the older women's knowledge and support for infant vaccination has been published in an earlier literature (24). Each older woman had at least 2 sessions of training before the infants were born. The pregnant women in the control arm had their infant vaccination as usual. The vaccination of all the infants were monitored from birth till 9 months and 2 weeks of age when all infant vaccination were expected to have been completed.

### Ethical considerations

The study protocol was approved by the Oyo State Ethics Research Committee and the University of Ibadan/University College Hospital Institutional Review Board. The permission to access the antenatal clinics was also obtained from the Coordinator of the PHC of Ibadan North and Ibadan North East local government areas (under the management of Oyo State Ministry of Health) where the PHCs were located. Both the pregnant women and the older women gave written informed consent before participation in the research. All identifiers like names and addresses were removed before data analysis to protect the identity of all participants.

### Data collection procedure

Data was obtained with the aid of a proforma using RedCap. Data about the sociodemographic characteristics of the pregnant women, the older women and the infants were collected. Visits were made to the homes of the women monthly and the date that each infant vaccine was received were documented as seen in the vaccination cards.

### Data analysis

Data was exported to Statistical Package for Social Sciences version 25 software. Socioeconomic class was determined using a method described earlier in a Nigerian study (25) where the socioeconomic class is a result of the composite score of the father and mothers' highest education attainments and their occupation. Descriptive analysis was used to generate frequencies and proportions for categorical variables such as socio demographic characteristics of the mothers, older women and infants. For this study, timeliness of a vaccine was defined as receipt of a vaccine within 2 weeks of the scheduled time. Also, vaccination completion was defined as receipt of four doses of oral polio vaccine (OPV), a single dose each of Bacille Calmette Guerin (BCG), Hepatitis B (HBV), measles, and yellow fever vaccine, and three doses of the Pentavalent (PENTA) vaccine (made up of Diptheria, Pertussis, Haemophilus influenza B and Hepatitis B antigens and Tetanus toxoid). Timeliness and completion of vaccination was also computed and presented in Tables. Association between selected independent variables and timeliness and completion of immunization was explored using the chi square test. All statistical significance was set at 5%.

## Results

A total of 211 pregnant women were recruited, however only 198 pregnant women had complete data post-intervention (105 in intervention group and 93 controls) and were included in the analysis. Their mean age was 27.6 ± 6.0 years. There were also 109 older women (mean age 55.8 ± 11.6 years. The details of the sociodemographic characteristics of the pregnant women and older women are as shown in Table 1. There were 202 infants (109 in intervention group and 93 controls) made up of 104(51.5%) males and 98(48.5%) females. All the babies in the intervention group were vaccinated but 2 in the control group did not receive any vaccine. Overall, about half of the infants (53.5%) had timely and complete vaccination and this was significantly higher in the intervention group compared with the controls. Specifically, the infants in the intervention group significantly had with timely and complete vaccination (67.9%) compared those in the control group (36.6%) (*p* < 0.01) as shown in Table 2. The *post hoc* power calculation showed that for a minimum sample size of 180 at 95% confidence with the prevalence of infants with timely and complete vaccination among the intervention and control group seen in the study being 0.7(67.9%) and 0.4 (36.6%), respectively gave a power of 90%.

**Table 1:** Socio-demographic characteristics of Mothers of infants and older women caregivers from selected slum communities in Ibadan, Nigeria.

**Selected Mothers'** **sociodemographic characteristics** **(*N* = 211)**	***n* (%)**	**Selected Older Women's** **sociodemographic characteristics** **(*N* = 109)**	***n* (%)**
**Age group (years)**		**Age group (years)**	
15–24	79(37.4)	35–44	15(13.8)
25–34	99(46.9)	45–54	31(28.4)
35–45	33(15.6)	55–64	35(32.1)
		>65	28(25.9)
**Education**		**Education**	
No formal	2(0.0)	No formal	29(26.6)
Primary	18(8.5)	Primary	22(20.4)
Secondary	165(78.2)	Secondary	54(50.0)
Tertiary	26(12.3)	Tertiary	4(3.7)
**Occupation**		**Occupation**	
Trading	74(35.1)	Trading	79(72.5)
Artisan	88(41.7)	Artisan	10(9.2)
Public worker	12(5.7)	Retired Public worker	5(4.6)
Patent Medicine Vendor	15(7.1)	Patent Medicine Vendor	1(0.9)
Unemployed	14(6.6)	Birth Attendant/Midwife	4(3.7)
Others	8(3.8)	Unemployed	10(9.2)
**Delay in ANC**		**Marital Status**	
Yes	130(61.9)	Married	81(75.0)
No	80(38.1)	Widowed	26(23.9)
		Single/Separated	2(1.8)
**Previous contraceptive use**		**Number of children had**	
Yes	61(30.7)	1–2	8(7.3)
No	138(69.3)	3–4	40(36.7)
		5 and above	61(56.0)
**Socio economic class**		**Relationship of older woman to mother**	
Low	46(21.8)	Mother-in-law	47(43.1)
Middle	154(73.0)	Mother	30(27.5)
High	11(5.2)	Sister	9(8.3)
		Sister-in-law	7(6.4)
		Aunt	6(5.5)
		Neighbor	6(5.5)
		Friend	2(1.8)
		Grandmother	2(1.8)

**Table 2:** Differences in vaccination timeliness and completion among infants in the intervention and control groups from selected urban slums communities in Ibadan, Nigeria.

**Study Arm**	**Timely and complete**	**Not timely nor complete**	**χ^2^**	***p*-value**
**Study Arm**				
Intervention	74(67.9)	35(32.1)		
Control	34(36.6)	59(63.4)	19.79	<0.01
**Total**	108(53.5)	94(46.5)		

In Table 3, the infants in the intervention group significantly received all the required vaccines at the right time compared with the infants in the control group. The BCG was the least timely of all the vaccines given at birth among the intervention group, while there was no difference in the timeliness of the same vaccine among the control group compared with other vaccines. Overall, the vaccines given at birth (BCG, OPV1, and HBV0) were less timely compared with other vaccines in both groups. There was an increase in the timeliness of the other vaccines (OPV1, 2 and 3; HBV1, 2 and 3; PENTA1, 2 and 3) subsequently in both groups except the last set of vaccines (measles, yellow fever and IM polio vaccines) given at 9 months of age that was slightly delayed in the control group.

**Table 3:** Timeliness of vaccines among intervention and control group of infants from selected slum communities in Ibadan, Nigeria.

**Vaccines**	**Intervention** ***n*(%)**	**Control** ***n*(%)**	**χ^2^**	***p*-value**
**BCG**				
Timely	100(91.0)	50(53.2)		
Not timely	8(7.3)	40 (42.6)	38.9	0.00
Not given	1(0.9)	4(4.3)		
**HBV0**				
Timely	101 (92.7)	50(53.2)		
Not timely	7(6.4)	40(42.6)	41.31	0.00
Not given	1(0.9)	4(4.3)		
**OPV0**				
Timely	101(92.7)	50(53.2)		
Not timely	7(6.4)	39(41.5)	41.27	0.00
Not given	1(0.9)	5(5.3)		
**OPV 1**				
Timely	105(96.3)	78(83.9)		
Not timely	4(3.7)	9(9.7)	10.7	0.00
Not given	0(0.0)	6(6.5)		
**PENTA 1**				
Timely	105(96.3)	78(83.9)		
Not timely	4(3.7)	9(9.7)	10.7	0.00
Not given	0(0.0)	6(6.5)		
**HBV 1**				
Timely	105(96.3)	78(83.9)		
Not timely	4(3.7)	9(9.7)	10.7	0.00
Not given	0(0.0)	6(6.5)		
**OPV 2**				
Timely	106(97.2)	78(84.8)		
Not timely	3(2.8)	9(9.8)	10.9	0.00
Not given	0(0.0)	5(5.4)		
**PENTA 2**				
Timely	106(97.2)	78(84.8)		
Not timely	3(2.8)	9(9.8)	10.9	0.00
Not given	0(0.0)	5(5.4)		
**HBV 2**				
Timely	106(97.2)	78(84.8)		
Not timely	3(2.8)	9(9.8)	10.9	0.00
Not given	0(0.0)	5(5.4)		
**OPV3**				
Timely	105(96.3)	73(79.3)		
Not timely	3(2.8)	13(14.1)	14.23	0.00
Not given	1(0.9)	6(6.5)		
**PENTA 3**				
Timely	105(96.3)	73(79.3)		
Not timely	3(2.8)	13(14.1)	14.23	0.00
Not given	1(0.9)	6(6.5)		
**HBV 3**				
Timely	105(96.3)	73(79.3)		
Not timely	3(2.8)	12(13.0)	14.31	0.00
Not given	1(0.9)	7(7.6)		
**Measles**				
Timely	90(97.8)	64(77.1)		
Not timely	2(2.2)	9(10.8)	18.43	0.00
Not given	0(0.0)	10(12.0)		
**Yellow fever**				
Timely	90(97.8)	63(76.8)		
Not timely	2(2.2)	9(11.0)	18.70	0.00
Not given	0(0.0)	10(12.0)		
**IM Polio**				
Timely	104(96.3)	71(78.9)		
Not timely	3(2.8)	13(14.4)	14.52	0.00
Not given	1(0.9)	6(6.7)		

BCG, Bacille Calmette Guerin; HBV, Hepatitis B vaccine; OPV, Oral Polio vaccine; PENTA, Pentavalent vaccine.

More infants whose older women caregivers were married significantly had timely and complete vaccination (75.3%) compared to those who were not married (45.8%) as shown in Table 4. Also, infants with older women who had 1 or 2 children had a higher proportion of timely and complete vaccination (85.7%) compared with those with five or more children (65.6%), though this was not statistically significant. Table 5 showed that male infants (59.6%) were more likely to have timely and complete vaccination compared to female infants (46.9%) (*p* = 0.25), and more infants whose birth weight were < 2.5 kg had both timely and complete vaccinations (71.4%) (*p* = 0.68). This proportion however reduced to 57.1% among those with normal birth weight and 41.2% in those with birth weight 3.5 kg and above (*p* > 0.05). In Table 6, infants with mothers aged < 25 years had a higher proportion (62.2%) of timely and complete vaccination and this same pattern was seen in both intervention and control groups. However, this was also not statistically significant. Unemployed mothers also had a higher proportion (76.9%) of infants who had timely and complete vaccination compared with other working mothers.

**Table 4:** Relationship between selected older women caregivers' socio demographic characteristics and overall timeliness and completeness of infant vaccination in urban slum communities of Ibadan, Nigeria.

**Selected sociodemographic characteristics**	**Timely and complete infant vaccination**			
	**Yes** ***n*(%)**	**No** ***n*(%)**	**Chi Square χ^2^**	***p*-value**
**Age group (years)**				
35–44	10(71.4)	4(28.6)		
45–54	20(64.5)	11(35.5)	2.28	0.51
55–64	27(77.1)	7(22.9)		
≥65	17(60.7)	11(39.3)		
**Marital status**				
Married	58(75.3)	19(24.7)		
Others	11(45.8)	13(54.2)	7.35	<0.01
**Education**				
No formal	20(71.4)	8(28.6)		
Primary	16(72.7)	6(27.3)	0.50	0.91
Secondary	35(66.0)	18(34.0)		
Tertiary	3(75.0)	1(25.0)		
**No of children**				
1–2	6(85.7)	1(14.3)		
3–4	28(70.0)	12(30.0)	1.24	0.53
5 and above	40(65.6)	21(34.4)		

**Table 5:** Relationship between infant characteristics and overall timeliness and completion of vaccination among infants in urban slum communities in Ibadan.

**Selected characteristics**	**Timely and complete**		**Chi square/ *p*-value**	**Not timely nor complete**		**Chi square/ *p*-value**
	**Intervention** ***n*(%)**	**Control** ***n*(%)**		**Intervention** ***n*(%)**	**Control** ***n*(%)**	
**Sex of child**						
Male	45(72.6)	17(27.4)	χ ^2^ = 1.29 *p* = 0.25	17(34.0)	25(59.5)	χ2 = 0.26 *p* = 0.60
Female	28(60.9)	18(39.1)		17(34.0)	33(66.0)	
**Birth weight**						
Less than 2.5 kg	3(60.0)	2(40.0)	χ^2^ = 0.22 *p* = 0.89	1(50.0)	1(50.0)	χ^2^ = 1.05 *p* = 0.59
2.5–3.5 kg	57(67.9)	27(32.1)		21(33.3)	42(66.7)	
>3.5 kg	10(71.4)	4(28.6)		9(45.0)	11(55.0)	
**Birth order**						
1	29(65.9)	15(34.1)	χ^2^ = 0.74 *p* = 0.68	12(37.5)	20(62.0)	χ^2^ = 0.12 *p* = 0.94
2–3	32(72.7)	12(27.3)		19(38.8)	30(61.2)	
4 and above	12(63.2)	7(36.8)		4(33.3)	8(66.7)	

**Table 6:** Relationship between selected mothers' socio demographic and overall timeliness of infant vaccination in urban slum communities in Ibadan.

**Selected characteristics**	**Timely and complete**		**Chi square/*p*-value**	**Not timely nor complete**		**Chi square/*p*-value**
	**Intervention** ***n*(%)**	**Control** ***n*(%)**		**Intervention** ***n*(%)**	**Control** ***n*(%)**	
**Age group**
15–24	34(73.9)	12(26.1)	χ^2^ = 1.14; *p* = 0.56	12(42.9)	16(57.1)	χ^2^ = 1.61 *p* = 0.42
25–34	32(65.3)	17(34.7)		14(30.4)	32(69.6)	
35–45	8(61.5)	8(38.5)		8(44.4)	10(55.6)	
**Occupation**
Business Woman/Trading	23(63.9)	13(36.1)	χ^2^ = 5.70 *p* = 0.33	12(36.4)	21(63.6)	χ^2^ = 7.10 *p* = 0.21
Artisan	33(71.7)	13(28.3)		14(36.8)	24(63.2)	
Civil servant	5(83.3)	1(16.7)		2(40.0)	3(60.0)	
Patent medicine vendor	2(40.0)	3(60.0)		3(30.0)	7(70.0)	
None	6(60.0)	4(40.0)		0(0.0)	3(100.0)	
Others	5(100.0)	0(0.0)		3(100.0)	0(0.0)	
**Education**
No formal	-	-	χ^2^ = 0.53 *p* = 0.76	1(50.0)	1(50.0)	χ^2^ = 3.19 *p* = 0.36
Primary	7(77.8)	2(22.2)		2(28.6)	5(71.4)	
Secondary	59(67.0)	29(33.0)		23(33.3)	46(66.7)	
Tertiary	8(72.7)	8(27.3)		8(57.1)	6(42.9)	
**Delay in ANC**
Yes	50(71.4)	20(28.6)	χ^2^ = 0.95 *p* = 0.32	21(41.2)	30(58.8)	χ^2^ = 0.87 *p* = 0.35
No	23(62.2)	14(37.8)		13(31.7)	28(68.3)	
**Previous contraceptive use**
No	50(71.4)	20(28.6)	χ^2^ = 1.39 *p* = 0.23	28(43.8)	36(56.2)	χ^2^ = 3.91 *p* = 0.04
Yes	21(60.0)	14(40.0)		5(20.8)	19(79.2)	
**Socio economic status**
Low	14(70.0)	6(30.0)	χ^2^ = 1.37 *p* = 0.50	5(27.8)	13(72.2)	χ^2^ = 0.85 *p* = 0.65
Middle	56(70.0)	24(30.0)		28(39.4)	43(60.6)	
High	4(50.0)	4(50.0)		1(33.3)	2(66.7)	

ANC, Antenatal care.

## Discussion

The training of older women caregivers about the importance of infant vaccination timeliness and completion greatly increased optimal infant vaccination in the intervention arm of this study. These infants significantly had timely and complete vaccination compared with the infants whose older women caregivers were not trained. This type of training appears promising in turning the tide of under-five mortality and morbidity arising from vaccine preventable diseases (VPDs) among infants of urban poor communities.

Many studies have highlighted the strong influence of older women in the care of children in the family and community (26, 27), with older women being viewed as custodians of traditional wisdom and cultural heritage in many cultures, especially in Africa (28, 29). We decided to leverage on the strategic position that these older women caregivers occupy in communities and families to improve infant vaccination timeliness and completion which have consistently been low in Nigeria among infant from families with the least wealth quintile. This similar picture is seen in many other developing countries (7, 30). Despite the earlier national interventions to improve infant vaccination coverage, there had been a slow improvement in infant vaccination rates and this was worse among those from lower socioeconomic class (31, 32), who also have lower rate in other health indices (33, 34) just as seen in the mothers' low uptake of contraceptives and antenatal care in this study.

The current intervention gives hope for the improvement of infant vaccination timeliness and completion in disadvantaged communities and has some edge over earlier interventions that were designed to achieve the same. The odds of its acceptance in other similar settings are high because it was built on an existing and respected cultural set up which community members could identify with. The focus here was on training older women caregivers who are influential and respected compared with general training of community members in an earlier intervention (35). This type of general training has been employed at different levels in Nigeria. The focused training employed in this study will reduce the resources required for the intervention. The skills that these women acquired can be transferred to others in their social network, broadening the impact. This will open the window for sustainability of the model as it will be easy for the communities to take ownership of the program. This is in addition to the contribution of cultural appeal and the fact that women were already traditionally involved in supervising infants' care. Therefore, there will be no need for the creation of new relationships or structure. This model can easily be adopted on a large scale because it will be cost effective compared with earlier interventions which used monetary and other forms of incentives to improve infant vaccination (36, 37) or those that adopted outreaches which are equally expensive (30). This is because aside the initial cost of the training, there will be very little subsequent cost, unlike the later interventions which will incur huge recurrent cost. The use of phone reminders (38) also required phone possession and electricity to keep the phone charged. These will cost money and might not be cost effective as compared with this model. This community based model has the capacity to have lager impact unlike facility based interventions (39) whose impact are likely to be restricted to some subset of the population.

The timeliness for each of the infant vaccines was significantly higher among the infants with trained older woman caregiver compared with those that were untrained. The importance of this is not only the effective protection from the VPDs that the vaccines target, but it will promote herd immunity and ensure that the infants do not become a threat to other infants if they become infected. They were also more likely to complete their vaccination as delayed vaccination have been linked with incomplete vaccination (21, 40). Specifically, timely BCG and HBV vaccination at birth will prevent tuberculosis and perinatal transmission of hepatitis B from mother to child respectively. These two diseases are endemic in many developing countries and prevention of transmission in infancy and beyond is important in their control.

Furthermore, the timeliness and completion of infant vaccination has been shown to depend on factors such as maternal age, education and level of income (40, 41). These factors were not significantly associated with timeliness and completion of infant vaccination in this study cohort. This makes the association of higher timeliness and completion of infant vaccination among infants with trained caregivers more relevant as it implies that the mothers' characteristics were not the responsible for the difference seen in the vaccination rate of both group of infants. The area of residence might explain the non-association of the women's characteristics with timeliness and completion of the infant vaccines as they were likely to share similar cultures and values which will in turn affect their perception and attitude toward infant vaccination (42). Schoeps et al. reported that timeliness and completion of BCG vaccination and completion of the vaccination schedule were affected by area of residence in Burkina Faso, suggesting that a community effect or belief may be at play in the decision to get the children vaccinated (41). A multinational study among sub-Saharan African countries also showed that living in poorer neighborhood was associated with incomplete vaccination (42). However, the training of the older women caregivers can also influence the mothers' perception about infant vaccination because of the strong influence of the older women. Further studies are required to see if this will be the case.

The timeliness and completeness of vaccination in this study was consistent all through the age of the infants among the intervention group in this study. This is in contrast with the typical pattern of gradual reduction in the timeliness and uptake of infant vaccination as the age of infants progress in sub-Saharan Africa (26, 40, 43). This is likely to be a reflection of the improved knowledge and efforts of the older women caregiver who ensured timely uptake of each vaccine by the infants. This further makes this model appealing as decline in vaccine uptake as an infant grows older have been a concern over the years in different settings. Distraction of the mother by other responsibilities looks like a plausible explanation for this reduction in timeliness and completion as the infant grows. The training and involvement of older women infant caregivers (where this set up exist) may be used to address this decline effectively, with resultant improvement in infant vaccination timeliness and completion.

It is important to look at some characteristics of the older women care givers which were associated with higher proportion of infants with timely and complete vaccinations. First, the older women care givers who were married might have the capacity to be more committed to overseeing the care of the infants they were assigned to because of the existing social support they enjoy as a result of their marital status. Being married in many African setting gives women access to more social support from family members who sometimes assist in carrying out domestic chores which are seen as primary responsibilities of women. This could create more room for the married women to engage in, and fare better in other activities like providing support for infant vaccination in their communities. Secondly, the reduction in domestic responsibilities could also explain the higher proportion of infants with timely and complete vaccination whose older women caregivers had two or less children. Such caregiver will have more time to provide care for an additional child (in this case, the infant) because of reduced demand for her time by her few children.

Gender Inequalities in immunization coverage have been shown to exist in many parts of the world (44). In this study, more male infants (72.6%) had timely and complete vaccination compared with the females (60.9%), although, this was not statistically significant. This has been reported by earlier researchers, typically in some developing countries where the male children were being given preferential treatments due to the patriarchal nature of the settings. An example is the report by Pande and Yazbeck where there was disparity in the vaccination rates of the infants in India with a higher male infant vaccination rates in 10 of the 17 major states in India (45). They noted that even states that performed well in immunization coverage struggled with considerably differences in immunization rates between boys and girls. In another Indian study, it was reported that girls had a 5% lower coverage compared to boys (46). This is likely to be the reason for the differential higher rate of under five mortality seen among female infants in some states in India (47). The reason for this gender difference in infant vaccination is not clear but it may be due to the cultural preference for the male child. Therefore, gender equity has to be addressed if the SDG 3 that aim to ensure health for people of all ages is to be achieved in Nigeria and in countries with similar discriminatory statistics.

There were also infant characteristics that had been associated with delay or incompletion of infant vaccination which were reversed in this study. For example, almost all the low birth weight (LBW) infants had both timely and complete vaccinations compared to normal weight infants. This is contrary to earlier reports where there were delayed vaccination of LBW infants (48, 49). This is a concern because mortality of LBW infants has the highest contribution to under five mortality and death from VPDs due to delayed vaccination will be an unnecessary and avoidable added cause of mortality among this group of infants. Urgent efforts are required to correct this. Our approach of involving older women caregivers in infant vaccination in communities needs to be tested among LBW infants at community level to determine how it can improve vaccination timeliness among these infants. There was a recent study in South Africa which demonstrated that it is possible to get LBW babies vaccinated in a timely manner (50) but the study was among LBW infants admitted in the hospital. Furthermore, a greater proportion of infants with higher birth order received timely and complete immunization which is in contrast to an earlier report (40). The reason for this is not clear and it will require further investigation.

It is interesting to note that some maternal factors which have been associated with delayed and incomplete infant vaccination in earlier reports also had reverse associations in this study. More infants of younger mothers had both timely and complete vaccination than their other counterparts and this is seen in both intervention and control groups. This contrasts with an earlier report where infants of older mothers were more likely to be fully vaccinated in Nigeria (32). Also, a higher proportion of infants of non-working mothers had timely vaccinations compared with those with working mothers (51–53). The working mothers in this study may have difficulty in balancing their time between work and the care of their infants. This is a vacuum that can be filled by the presence of a trained older woman caregiver who can step in to ensure the infants are vaccinated even when the mothers are busy at work. These contrary associations need to be further investigated to establish if the results were solely due to the training of the older women caregivers.

This study has some strengths and this includes the novel and low cost strategy of training of older women caregivers which builds on an existing structure in the community. This provides specific solution to the suboptimal vaccination of infants in urban slums which has defied solutions by the current strategies to promote optimal infant vaccination. Its short and long term impacts require further study. The odds of the sustainability of this strategy are high because it was built on the people's culture and it was community based. There is likely to be spillover of the skills learnt in the social circles of the trained older women.

There were a number of limitations in this study as well. The strategy is only relevant in settings where older women are involved in the care of infants. It may also have a different effect on infant vaccination if it is used outside urban slums because of the differences in the education and economic status of mothers in such settings.

## Conclusion

In conclusion, the involvement of trained older women caregivers in infant vaccination improved both the timeliness and completion of infants' vaccination in urban slum. There were evidences that the training also increased the timeliness and completion of vaccination of infants who were LBW and those with higher birth order, contrary to earlier reports. This appears to be a promising strategy to improve the quality of infant vaccination in disadvantaged communities where older women oversee the care of infants. However, this strategy still needs to be tested in a larger population to firmly establish its impacts on infant vaccination timeliness and completion.

## Data availability statement

The datasets presented in this study can be found in online repositories. The name of the repository and accession number can be found at: Mendeley Data, V1, doi: 10.17632/g8cnrk58mv.1.

## Ethics statement

The study protocol was approved by the Oyo State Ethics Research Committee and the University of Ibadan/University College Hospital Institutional Review Board. The permission to access the antenatal clinics was also obtained from the Coordinator of the PHC of Ibadan North and Ibadan North East local government areas (under the management of Oyo State Ministry of Health) where the clinics were located. Both the pregnant women and the older women gave written informed consent before participation in the research. The mothers also gave consent for their infants' participation.

## Author contributions

FB conceived the research idea and coordinated the data collection. FB and AA designed the study while EB analyzed the data. All the authors interpreted the results, wrote the manuscript and approved its final draft, and attest they meet the ICMJE criteria for authorship.

## Funding

This project was supported by the Bill and Melinda Gates Foundation under the Grand Challenge Explorations [OPP1190834]. The funder had no role in the design of the study, data collection, analysis and interpretation, as well as the writing of the manuscript.

## Conflict of interest

The authors declare that the research was conducted in the absence of any commercial or financial relationships that could be construed as a potential conflict of interest.

## Publisher's note

All claims expressed in this article are solely those of the authors and do not necessarily represent those of their affiliated organizations, or those of the publisher, the editors and the reviewers. Any product that may be evaluated in this article, or claim that may be made by its manufacturer, is not guaranteed or endorsed by the publisher.
